# Psychological resilience moderates the association between adverse academic events and probable anxiety disorder in Chinese clinical medicine postgraduates: a cross-sectional study

**DOI:** 10.1186/s12909-026-09761-z

**Published:** 2026-06-19

**Authors:** Lei Huang, Jiahui Kou, Yiwen Zhang, Nian Chen, Jin Chen, Xiaomin Zhou, Yan Wu

**Affiliations:** Department of Postgraduate Students, West China School of Medicine, West China Hospital, Sichuan University, No. 37 Guoxue Alley, Wuhou District, Chengdu, Sichuan Province P.R. China

**Keywords:** Medical education, Clinical medicine postgraduates, Adverse academic events, Probable anxiety disorder, Psychological resilience, Moderating effect

## Abstract

**Background:**

Clinical medicine postgraduates face a heightened risk for mental health problems, particularly anxiety disorder, which is largely due to a high-pressure training environment and prevalent adverse academic events. Psychological resilience, defined as the ability to adapt to adversity, is theorized to modify the association of external stressors with mental health problems. However, limited evidence exists regarding its moderating role in this specific population, particularly using rigorous interaction analysis.

**Methods:**

In this cross-sectional study, 1182 clinical medicine postgraduates from a major Chinese medical school completed an anonymous online survey. Exposure to adverse academic events was assessed via an 8-item checklist. Anxiety symptoms were measured using the Generalized Anxiety Disorder scale (GAD-7), and psychological resilience was assessed with the Connor-Davidson Resilience Scale (CD-RISC-10). Hierarchical logistic regression was used to examine associations. The moderating role of psychological resilience was tested using the interaction contrast approach, reporting the Relative Excess Risk due to Interaction (RERI), Attributable Proportion (AP), and Synergy Index (SI).

**Results:**

Reporting adverse academic events was strongly associated with probable anxiety disorder (OR = 3.157, 95% CI: 2.284–4.363, *p* < 0.001), and low psychological resilience was independently associated with higher odds (OR = 3.657, 95% CI: 2.546–5.253, *p* < 0.001). A significant additive interaction was observed (RERI = 5.308, 95% CI: 1.402–9.214; AP = 0.434, 95% CI: 0.218–0.650; SI = 1.897, 95% CI: 1.218–2.955). Stratified analysis revealed that the association of adverse academic events with probable anxiety disorder was substantially stronger among individuals with low resilience (OR = 12.226, 95% CI: 7.585–19.708) compared to those with high resilience (OR = 3.775, 95% CI: 2.043–6.975).

**Conclusions:**

Adverse academic events are positively associated with probable anxiety disorder among clinical medicine postgraduates. Psychological resilience is a potent factor that modifies this association. These findings highlight the importance of integrating resilience assessment and resilience‑building programs into postgraduate medical education to safeguard trainee mental well-being.

## Background

Postgraduates are a population particularly susceptible to mental health issues [1]. Due to high academic demands and social expectations, coupled with a series of uncertainties regarding career planning and future development, this group faces dual pressure on both physiological and psychological levels [2, 3]. Clinical medicine postgraduates represent a critical cohort within the healthcare training pipeline, yet they face exceptionally high academic, clinical, and research demands [4–6]. A narrative qualitative systematic review encompassing over half a million medical students identified academic pressure as a predominant stressor [7]. The intense training environment, characterized by long working hours, high-stakes examinations, publication pressures, and frequent encounters with patient suffering, places this group at heightened risk for psychological distress, particularly anxiety disorder and depression [8–10]. Left unaddressed, impaired mental well-being not only compromises trainees’ personal health but may also affect clinical performance, professional development, and ultimately, patient care [11].

Adverse academic events, such as experimental failure, manuscript rejection, conflict with supervisors, or failing qualifying exams, are common and potent stressors during postgraduate training [12–14]. A growing body of evidence highlights that such events are significantly associated with increased psychological distress, including but not limited to anxiety and depressive symptoms, among medical postgraduates [15–18]. However, most existing studies have focused on documenting the direct association between academic stressors and anxiety, often treating stress exposure as a uniform risk factor without sufficiently accounting for individual differences in coping resources.

Psychological resilience, defined as the ability to adapt positively to adversity, has been recognized as a key internal resource that is associated with a weaker negative psychological response to stressors [19, 20]. Rooted in the conservation of resources model [21], resilience helps individuals maintain emotional stability and functional competence under pressure [22, 23]. In various high-stress populations, higher resilience has been linked to lower levels of anxiety and depression [24, 25]. Nevertheless, in the context of clinical medicine postgraduate training, relatively few studies have quantitatively examined whether resilience modifies the relationship between academic adversity and anxiety. To address this gap, the present study proposes a mechanistic framework for how resilience may modify this association specifically. First, resilient individuals tend to appraise academic setbacks (e.g., manuscript rejection, experimental failure) as less threatening and more controllable, thereby reducing the perceived severity of the stressor [26]. Second, resilience is associated with greater use of adaptive coping strategies and less reliance on maladaptive strategies [27]. These coping processes directly influence the phenotypic expression of anxiety, for example, by lowering the frequency and intensity of worry, reducing physiological hyperarousal, and preventing the escalation of transient anxiety into persistent, clinically significant symptoms [28]. Third, resilience promotes the maintenance of positive affect even under prolonged pressure, which counteracts the emotional exhaustion that often accompanies academic stress [29]. Notably, these modifying effects may be more pronounced for academic stressors than for general life stressors, because academic adversity is often chronic, role‑specific, and directly tied to the professional identity. Resilience helps preserve a sense of competence and purpose in the face of academic failure, thereby attenuating the specific anxiety triggered by threats to one‘s academic self‑concept [30]. Understanding this specificity is critical for designing targeted interventions that strengthen resilience in medical education settings [31].

A notable research gap remains: while several studies report main effects of either academic stressors or resilience on mental health outcomes, few have applied formal interaction analysis, particularly on the additive scale, to test whether resilience significantly attenuates the effect of academic stressors on anxiety among clinical medicine postgraduates. Most prior work relies on correlation or stratified descriptive approaches, which do not formally quantify the magnitude of effect modification. This limits the ability to determine whether enhancing resilience could meaningfully reduce the anxiety risk associated with academic adversity.

Therefore, this study aimed to investigate the association between adverse academic events and probable anxiety disorder among clinical medicine postgraduates, and to examine whether psychological resilience moderates this relationship. It was hypothesized that: (H1) adverse academic events would be positively associated with probable anxiety disorder; (H2) higher psychological resilience would be negatively associated with probable anxiety disorder; and (H3) psychological resilience would significantly modify the association between adverse academic events and probable anxiety disorder, such that the association of adverse academic events with probable anxiety disorder would be weaker among individuals with high resilience.

## Methods

### Study design and ethical approval

This study employed a cross-sectional design. Reporting was presented based on the STROBE checklist. The study protocol was reviewed and approved by the Ethics Committee of West China Hospital, Sichuan University (Approval No. 2019 − 980). Electronic informed consent was obtained from all participants prior to survey commencement.

### Sample size, participants and setting

**Fig. 1:**
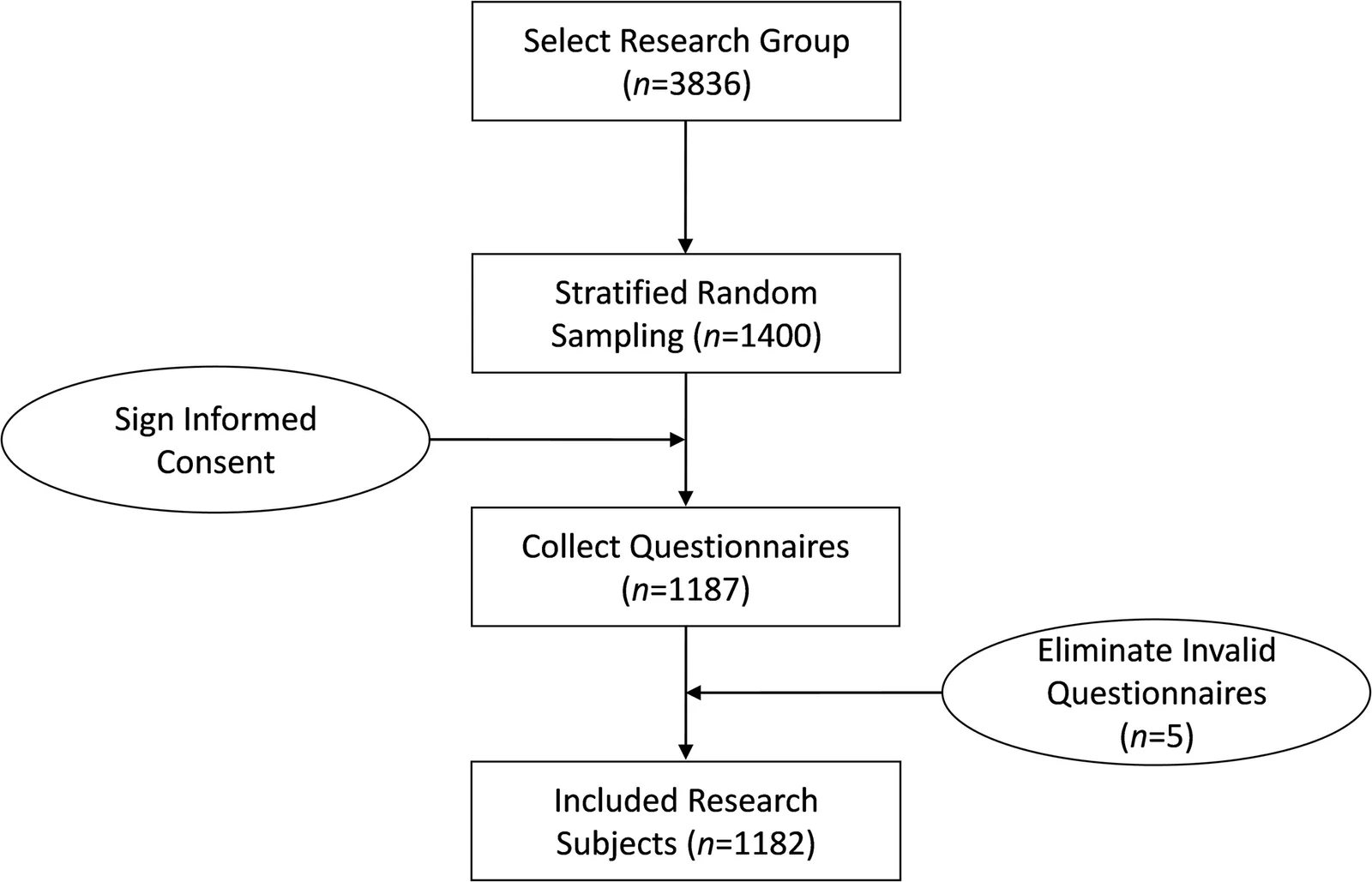
Flow diagram of the research subjects

Data were collected between June and November 2025 through an anonymous online survey hosted on the Wenjuanxing platform. A stratified random sampling method was utilized to recruit clinical medicine postgraduates from the West China School of Medicine, Sichuan University. Inclusion criteria were: (1) full-time enrollment; (2) current enrollment in a clinical medicine master’s or doctoral program; and (3) provision of informed consent to participate. Exclusion criteria included: (1) part-time enrollment or international students; (2) first-year postgraduates (who had not yet begun academic/clinical rotations); and (3) students on a leave of absence. A total of 1400 potential participants were initially identified. Of these, 1187 completed the survey, yielding a response rate of 84.8%. After excluding 5 participants with incomplete data on any of the core questionnaires (anxiety disorder scale, resilience scale, and adverse academic events checklist), 1182 respondents were included in the final analysis. A flowchart detailing the participant inclusion process is presented in Fig. 1.

### Measures

#### Demographic and training-related variables

Participants reported age, sex, program duration (3-year, 4-year, or 5-year program), student category (academic master’s degree, professional master’s degree, academic doctorate, or professional doctorate), frequency of long workdays (≥ 10 h per day), night shift frequency, monthly income, and physical exercise frequency.

#### Adverse academic events

Exposure to adverse academic events was investigated using an 8‑item self‑developed checklist. The items were generated based on a review of the literature and semi‑structured interviews with clinical medicine postgraduates, and were designed to capture common stressors encountered during postgraduate training. The checklist included four academic‑specific adversities (“experimental failure”, “manuscript rejection”, “conflict with supervisor”, “failure in qualifying exams”); and four other major life stressors (“serious medical error/accident”, “personal severe illness/injury”, “serious illness of a family member”, “break‑up of a romantic relationship”). To ensure content validity, the checklist was piloted with 30 clinical medicine postgraduates prior to the main survey. Feedback was used to clarify wording and confirm that the academic events were relevant and comprehensively represented the target stressor domain. Participants indicated whether they had experienced each event in the past 12 months (yes/no). For the primary analysis, respondents were dichotomized into a “no-event-reported group” (no academic‑specific event endorsed) and an “event-reported group” (at least one academic‑specific event endorsed). Other major life stressors were recorded but not included in the definition of the primary exposure variable. This approach ensured that the analysis specifically captured the effect of academic adversity rather than general life stressors.

#### Anxiety symptoms

Symptoms of generalized anxiety disorder were measured using the Chinese version of the 7-item Generalized Anxiety Disorder scale (GAD-7) [32, 33]. Respondents rate the frequency of each symptom over the past two weeks on a 4-point Likert scale ranging from 0 (not at all) to 3 (nearly every day). Total scores range from 0 to 21, with a cut-off score of ≥ 10 indicating probable clinical levels of anxiety. The GAD-7 has demonstrated good reliability and validity in Chinese populations. In this study, its Cronbach’s α was 0.95.

#### Psychological resilience

Psychological resilience was assessed using the Chinese version of the 10-item Connor-Davidson Resilience Scale (CD-RISC-10) [34, 35]. Items are rated on a 5-point scale from 0 (not true at all) to 4 (true nearly all the time). Total scores range from 0 to 40, with higher scores indicating greater psychological resilience. The scale has shown good psychometric properties in previous studies. In the present sample, its internal consistency was very high (Cronbach’s α = 0.97). For logistic regression analysis and interaction analysis, a median split (score > 27 vs. ≤ 27) was used to define high-resilience and low-resilience group. As no clinically validated cut‑off for the CD-RISC‑10 exists in this population, the median split ensures balanced group sizes and maximizes statistical power for detecting interaction effects.

### Statistical analysis

All statistical analyses were conducted using STATA software (version 14.0), with a two-sided significance level (α) set at 0.05. Descriptive statistics were computed for all study variables. The Kolmogorov‒Smirnov normality test was used to assess the distribution of GAD-7 scores. Differences in GAD-7 scores across subgroups defined by basic characteristics were assessed using Mann-Whitney test (two groups) and Kruskal‒Wallis test (multiple groups). Spearman’s rank-order partial correlation was used to examine the associations among the main study variables. To identify factors associated with probable anxiety disorder, hierarchical logistic regression was employed. The analysis proceeded in three sequential models: Model 1 included demographic variables; Model 2 added lifestyle and training-related covariates; and the final model (Model 3) introduced the primary explanatory variables, adverse academic events and psychological resilience. Odds ratios (ORs) with 95% confidence intervals (CIs) were reported. Multicollinearity was checked using the variance inflation factor (VIF); all values were < 5, confirming no substantial collinearity among the covariates.

The moderating role of psychological resilience in the relationship between adverse academic events and probable anxiety disorder was assessed by testing for interaction [36]. This was operationalized using the interaction contrast approach for biological interaction. In a 2 × 2 table classifying exposure to adverse academic events (yes/no) and level of psychological resilience (high/low), the presence of an additive interaction was indicated if the combined odds ratio (OR_11_) exceeded the sum of the individual odds ratios minus one (OR_10_ + OR_01_ − 1), while a multiplicative interaction was indicated if OR_11_ exceeded the product of the individual odds ratios (OR_10_ × OR_01_) [37]. This interaction was quantified using three key indices: the Relative Excess Risk due to Interaction (RERI = OR_11_ - OR_10_ - OR_01_ + 1), the Attributable Proportion (AP = RERI / OR_11_), and the Synergy Index (SI = (OR_11_ − 1) / (OR_10_ + OR_01_ − 2) under an additive model; SI = OR_11_ / (OR_10_ × OR_01_) under a multiplicative model). The SI greater than 1 indicates a positive interaction or synergy between the factors [36, 38].

## Results

### Participant characteristics

A total of 1182 clinical medicine postgraduates participated in this study. The sample characteristics are presented in Table 1. The median age of participants was 26 (Q1–Q3: 25–28) years, and 65.5% (*n* = 774) were female. The distribution across degree programs was as follows: 25.0% academic master’s degree, 27.3% professional master’s degree, 29.0% academic doctorate, and 18.7% professional doctorate. A significant proportion (36.4%) reported working long hours (≥ 10 h per day) for 6–7 days per week, and 31.0% reported never engaging in physical exercise. Overall, 257 participants (21.7%) had GAD-7 scores ≥ 10, indicating probable anxiety disorder. The Kolmogorov-Smirnov normality test showed that the GAD-7 scores did not conform to the normal distribution (*p* < 0.05). The group reporting adverse academic events (40.5%, *n* = 479) had significantly higher GAD-7 scores (median 7.0 [6.0–12.0]) than the no-event-reported group (median 5.0 [1.0–7.0], *p* < 0.001). Similarly, the low-resilience group (CD-RISC-10 score ≤ 27, 52.7%, *n* = 623) reported significantly higher GAD-7 scores (median 7.0 [6.0–12.0]) than the high-resilience group (median 3.0 [1.0–7.0], *p* < 0.001). Statistically significant differences in GAD-7 scores were also found across categories of student category, frequency of long workdays, monthly income, and physical exercise (all *p* < 0.05).

**Table 1:** Basic characteristics of the research subjects

Variable and category		GAD-7 scores		
		*N* (%)	Median (Q1–Q3)	*p* Value
Age (year)	< 25	286 (24.2)	7.0 (3.0–9.0)	0.153
	25–29	727 (61.5)	7.0 (3.0–8.0)	
	≥ 30	169 (14.3)	6.0 (2.0–7.0)	
Sex	male	408 (34.5)	7.0 (2.0–8.0)	0.564
	female	774 (65.5)	7.0 (3.0–8.0)	
Program duration	3-year program	840 (71.1)	7.0 (3.0–9.0)	0.264
	4-year program	278 (23.5)	6.0 (2.0–7.0)	
	5-year program	64 (5.4)	6.5 (3.0–9.0)	
Student category	academic master’s degree	295 (25.0)	7.0 (3.0–9.0)	0.001
	professional master’s degree	323 (27.3)	7.0 (4.0–10.0)	
	academic doctorate	343 (29.0)	6.0 (2.0–7.0)	
	professional doctorate	221 (18.7)	6.0 (2.0–7.0)	
Frequency of long workdays (≥ 10 h)	0–3 days per week	354 (30.0)	6.0 (2.0–7.0)	< 0.001
	4–5 days per week	397 (33.6)	6.0 (2.0–7.0)	
	6–7 days per week	431 (36.4)	7.0 (4.0–10.0)	
Night shift frequency	never	769 (65.0)	6.0 (2.0–8.0)	0.040
	occasionally	44 (3.7)	6.0 (3.0–7.0)	
	once a week	211 (17.9)	7.0 (3.0–9.0)	
	2 days a week or more	158 (13.4)	7.0 (4.0–10.0)	
Income (RMB)	≥ 4000	198 (16.8)	6.0 (2.0–7.0)	0.031
	2000–3999	575 (48.6)	7.0 (3.0–8.0)	
	< 2000	409 (34.6)	7.0 (3.0–9.0)	
Physical exercise frequency	never	366 (31.0)	7.0 (5.0–11.0)	< 0.001
	occasionally	423 (35.8)	6.0 (2.0–7.0)	
	1–2 times a week	250 (21.1)	6.0 (2.0–7.0)	
	3 times a week or more	143 (12.1)	6.0 (2.0–7.0)	
Adverse academic events	no-event-reported group	703 (59.5)	5.0 (1.0–7.0)	< 0.001
	event-reported group	479 (40.5)	7.0 (6.0–12.0)	
Psychological resilience	high-resilience group (> 27)	559 (47.3)	3.0 (1.0–7.0)	< 0.001
	low-resilience group (≤ 27)	623 (52.7)	7.0 (6.0–12.0)	

GAD-7, 7-item Generalized Anxiety Disorder scale. GAD-7 scores are not normally distributed and are summarized as median (Q1–Q3). Group comparisons are based on Kruskal–Wallis or Mann–Whitney U tests

### Associations between study variables

**Fig. 2:**
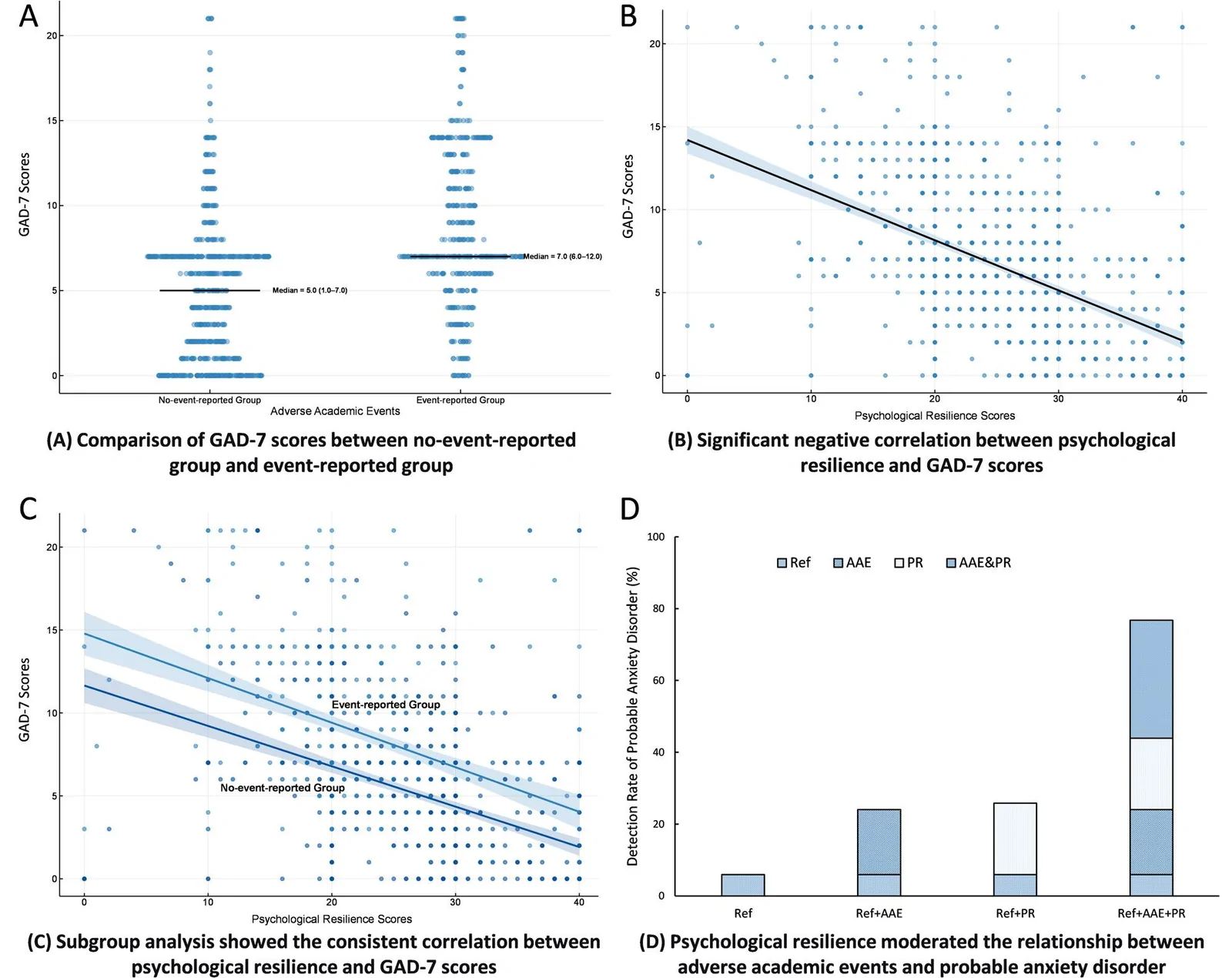
Association of adverse academic events and psychological resilience with probable anxiety disorder. Note: GAD-7, 7-item Generalized Anxiety Disorder Scale. AAE, Adverse Academic Events. PR, Psychological Resilience. **A** The group reporting adverse academic events had significantly higher GAD-7 scores than the no-event-reported group. **B** Psychological resilience was significantly negatively correlated with GAD-7 scores. **C** The significant negative correlation remained consistent across subgroups defined by adverse academic events. **D** Psychological resilience significantly moderated the relationship between adverse academic events and probable anxiety disorder

Spearman’s rank-order partial correlation was conducted to examine the relationships between key study variables and GAD-7 scores (Table 2). Adverse academic events showed a significant positive correlation with GAD-7 scores (partial *r* = 0.259, *p* < 0.001). In contrast, psychological resilience was significantly negatively correlated with GAD-7 scores (partial *r* = -0.450, *p* < 0.001). Among the covariates, a higher frequency of long workdays (≥ 10 h) was positively associated with GAD-7 scores (partial *r* = 0.095, *p* = 0.001), while more frequent physical exercise showed a negative association (partial *r* = -0.066, *p* = 0.025). These correlational patterns were visually supported by the presented scatter plots and line graphs, which illustrated the same directional associations and trends between adverse academic events, psychological resilience, and GAD-7 scores (Fig. 2A, B, and C).

**Table 2:** Spearman’s rank-order partial correlation among the study variables

Variable	Partial Corr.	Semipartial Corr.	Partial Corr.^2	Semipartial Corr.^2	*p* Value
Age (year)	-0.025	-0.020	0.001	0.000	0.388
Sex	-0.041	-0.033	0.002	0.001	0.164
Program duration	0.035	0.028	0.001	0.001	0.232
Student category	-0.028	-0.023	0.001	0.001	0.332
Frequency of long workdays (≥10 h)	0.095	0.077	0.009	0.006	0.001
Night shift frequency	0.062	0.050	0.004	0.003	0.034
Income (RMB)	0.053	0.042	0.003	0.002	0.072
Physical exercise frequency	-0.066	-0.053	0.004	0.003	0.025
Adverse academic events	0.259	0.215	0.067	0.046	<0.001
Psychological resilience	-0.450	-0.404	0.202	0.163	<0.001

### Logistic regression analysis

Hierarchical logistic regression was performed to identify factors associated with probable anxiety disorder (Table 3). Model 1 included basic demographics, which were not significant predictors. Model 2 added lifestyle and training-related factors. In this model, monthly income (2000–3999 RMB: OR = 1.815, 95% CI = 1.142–2.885, *p* = 0.012; < 2000 RMB: OR = 2.847, 95% CI = 1.606–5.048, *p* < 0.001) and frequency of physical exercise (occasionally: OR = 0.440, 95% CI = 0.311–0.621, *p* < 0.001; 1–2 times a week: OR = 0.411, 95% CI = 0.271–0.623, *p* < 0.001; 3 times a week or more: OR = 0.467, 95% CI = 0.280–0.779, *p* = 0.004) were significantly associated with probable anxiety disorder. In the final model (Model 3), both primary variables were entered. Reporting adverse academic events was associated with higher odds of probable anxiety disorder (OR = 3.157, 95% CI = 2.284–4.363, *p* < 0.001). Similarly, low psychological resilience was also associated with higher odds (OR = 3.657, 95% CI = 2.546–5.253, *p* < 0.001), indicating that individuals with low psychological resilience had 3.657 times higher odds of probable anxiety disorder.

**Table 3:** Logistic regression of adverse academic events, psychological resilience and probable anxiety disorder

Model and variable		Probable anxiety disorder	
		OR (95% CI)	*p* Value
Model 1			
Age (year)	< 25	Ref	
	25–29	1.065 (0.744, 1.524)	0.732
	≥ 30	1.370 (0.777, 2.415)	0.276
Sex	male	Ref	
	female	1.027 (0.763, 1.382)	0.862
Program duration	3-year program	Ref	
	4-year program	0.406 (0.048, 3.418)	0.407
	5-year program	0.632 (0.069, 5.791)	0.684
Student category	academic master’s degree	Ref	
	professional master’s degree	1.214 (0.837, 1.761)	0.308
	academic doctorate	1.646 (0.190, 14.233)	0.651
	professional doctorate	0.673 (0.408, 1.109)	0.120
Model 2			
Frequency of long workdays (≥ 10 h)	0–3 days per week	Ref	
	4–5 days per week	0.940 (0.644, 1.371)	0.748
	6–7 days per week	1.363 (0.951, 1.955)	0.092
Night shift frequency	never	Ref	
	occasionally	0.901 (0.372, 2.182)	0.817
	once a week	1.035 (0.596, 1.797)	0.904
	2 days a week or more	1.300 (0.741, 2.281)	0.361
Income (RMB)	≥ 4000	Ref	
	2000–3999	1.815 (1.142, 2.885)	0.012
	< 2000	2.847 (1.606, 5.048)	< 0.001
Physical exercise frequency	never	Ref	
	occasionally	0.440 (0.311, 0.621)	< 0.001
	1–2 times a week	0.411 (0.271, 0.623)	< 0.001
	3 times a week or more	0.467 (0.280, 0.779)	0.004
Model 3			
Adverse academic events	no-event-reported group	Ref	
	event-reported group	3.157 (2.284, 4.363)	< 0.001
Psychological resilience	high-resilience group (> 27)	Ref	
	low-resilience group (≤ 27)	3.657 (2.546, 5.253)	< 0.001

Model 2 adjusted for age, sex, program duration, student category. Model 3 adjusted for age, sex, program duration, student category, frequency of long workdays, night shift frequency, income, physical exercise frequency

### Moderating role of psychological resilience

The moderating effect of psychological resilience on the relationship between adverse academic events and probable anxiety disorder was assessed using measures of additive and multiplicative interaction (Table 4). The odds ratio for the joint effect of adverse academic events and psychological resilience (OR_11_) was 12.226 (95% CI: 7.585–19.708). The individual effect of adverse academic events (OR_01_) was 3.775 (95% CI: 2.043–6.975), and the individual effect of psychological resilience (OR_10_) was 4.144 (95% CI: 2.467–6.961). The joint effect OR_11_ exceeded OR_10_ + OR_01_ − 1 (6.919), suggesting a potential additive interaction, but was lower than OR_10_ × OR_01_ (15.644), indicating no multiplicative interaction. The RERI was 5.308 (95% CI: 1.402–9.214, *p* = 0.008), the AP was 0.434 (95% CI: 0.218–0.650, *p* < 0.001), and the SI was 1.897 (95% CI: 1.218–2.955, *p* = 0.005). Stratified analysis (Table 4; Fig. 2D) further illustrated the moderating effect. Among individuals with high psychological resilience, exposure to adverse academic events increased the odds of probable anxiety disorder (OR = 3.775, 95% CI = 2.043–6.975). However, this effect was substantially magnified among low-resilience group (OR = 12.226, 95% CI = 7.585–19.708). This indicates that the association between adverse academic events and probable anxiety disorder was significantly stronger among individuals with low psychological resilience.

**Table 4:** Moderating effect of psychological resilience on the relationship between adverse academic events and probable anxiety disorder

Adverse academic events	Psychological resilience			
	high-resilience group (scores > 27)		low-resilience group (scores ≤ 27)	
	*N*(Case/Control)	OR(95% CI)	*N*(Case/Control)	OR (95% CI)
no-event-reported group	24/406	1	56/217	4.144 (2.467, 6.961)
event-reported group	25/104	3.775 (2.043, 6.975)	152/198	12.226 (7.585, 19.708)
ORs (95% CI) stratified by psychological resilience	4.072 (2.139, 7.751) *p* < 0.001		2.902 (1.986, 4.241) *p* < 0.001	

RERI = 5.308 (1.402, 9.214), *P* = 0.008; AP = 0.434 (0.218, 0.650), *P* < 0.001; SI = 1.897 (1.218, 2.955), *P* = 0.005; ORs adjusted for age, sex, program duration, student category, frequency of long workdays, night shift frequency, income, physical exercise frequency

## Discussion

### Summary of key findings

This study examined the association between adverse academic events and probable anxiety disorder among clinical medicine postgraduates in China, and tested the moderating role of psychological resilience. The findings supported all three hypotheses. First, reporting adverse academic events was significantly associated with higher odds of probable anxiety disorder. Second, lower psychological resilience was independently and strongly associated with probable anxiety disorder. Most importantly, this study found a significant additive interaction, indicating that psychological resilience significantly moderated the relationship. The association between adverse academic events and probable anxiety disorder was substantially stronger among individuals with low psychological resilience, whereas it was markedly weaker among those with high resilience.

### Comparison with existing literature and interpretation

The positive association between adverse academic events and probable anxiety disorder is consistent with a large body of literature documenting the high prevalence of mental distress among medical trainees [39–41]. This study adds specificity by focusing on discrete, adverse academic events, which are actionable targets for institutional support. The strong, inverse relationship between psychological resilience and probable anxiety disorder aligns with the core tenet of resilience theory and corroborates findings from other high-stress professional groups [42, 43], suggesting that resilience as a universal key resource. The central contribution of this study lies in the formal quantification of the modifying effect of resilience. While the modifying role of resilience is often assumed, few studies in medical education have empirically tested this interaction using additive measures. The significant RERI and AP values indicate that a substantial portion of the higher anxiety odds among those exposed to both low resilience and adverse academic events is attributable to their synergistic interaction, not merely the sum of their individual effects. This synergy can be interpreted through the lens of the conservation of resources theory [21, 23, 44]. Individuals with low resilience may possess fewer psychological resources to cope with academic setbacks, which is associated with more rapid resource depletion and heightened anxiety. In contrast, those with high resilience can draw upon a reservoir of adaptive resources, which corresponds to a weaker stressor‑anxiety relationship.

### Implications for medical education practice

These findings have direct, actionable implications for postgraduate medical education. Firstly, institutions should consider integrating routine, anonymized screening for both academic stress and psychological resilience into student support systems. Identifying students with low resilience and high exposure to stressors can enable targeted, early intervention. Secondly, resilience is a malleable trait. Medical schools should prioritize the development and implementation of evidence-based resilience training programs, as such programs have demonstrated efficacy in boosting psychological resilience and reducing anxiety [45]. These could be embedded within the curriculum as mandatory well-being modules, covering skills such as cognitive reframing, emotional regulation, mindfulness, and stress management techniques. Thirdly, faculty development is crucial. Training supervisors and mentors to recognize signs of distress, understand the role of resilience, and provide psychologically informed support can foster a more supportive training climate. Finally, facilitating peer support networks can create an additional protective layer, allowing students to share experiences and coping strategies.

Beyond these individual‑level strategies, the findings also point to the need for institutional‑level interventions that address the high‑stakes academic culture itself. The strong association between adverse academic events and probable anxiety disorder suggests that simply teaching students to “bounce back” may be insufficient if the training environment remains chronically stressful. Institutions should therefore consider reducing unnecessary achievement pressure, reforming evaluation systems that overemphasize publication quantity, providing structured mentorship to mitigate academic setbacks, and fostering a learning climate where failure is normalized as part of professional growth rather than stigmatized. Such environmental modifications could lower the baseline exposure to academic adversity, thereby lessening the demand for individual resilience.

### Strengths and limitations

This study has several strengths, including a large, well-defined sample of clinical medicine postgraduates, the use of validated instruments (GAD-7, CD-RISC-10), and the application of interaction contrast (biological interaction) to quantify effect modification on the additive scale (RERI, AP, SI), which is increasingly recommended for public health and clinical interpretation. Several limitations must be acknowledged. (1) The cross-sectional design precludes causal inference. While it is hypothesized that adverse academic events erode mental health, it is also possible that pre-existing anxiety influences the perception or reporting of events. The 12-month recall period does not allow determination of whether pre-existing anxiety symptoms preceded the reported adverse academic events; the absence of baseline anxiety assessment before the recall period precludes causal inferences regarding the temporal sequence. (2) Data were self-reported, which may introduce recall or social desirability bias. The sample was drawn from a single, albeit large, medical school in China, which may limit the generalizability of findings to other cultural or educational contexts. (3) Clinical specialty type was not adjusted for due to the risk of model overfitting, which is also acknowledged as a limitation. Furthermore, other potential moderators, such as social support or personality traits, were not accounted for, and these could also influence the observed relationships. (4) The outcome, probable anxiety disorder, had a relatively high prevalence in this study (approximately 37% among the event‑exposed group). Consequently, the odds ratios derived from logistic regression may overestimate the corresponding risk ratios, and the OR‑based interaction indices (RERI, AP, SI) should be interpreted on the odds scale rather than as measures of risk or risk difference.

### Future research

Future studies should employ longitudinal designs to delineate the temporal and potentially reciprocal relationships between academic stress, resilience, and anxiety over the course of postgraduate training. From a transdiagnostic perspective, the modifying role of resilience observed in this study is likely not limited to anxiety alone, as resilience has been associated with depression, burnout, and other stress‑related conditions. Future research should explicitly test the transdiagnostic efficacy of resilience‑focused interventions. Given the current scarcity of high-quality evidence specifically for healthcare students [46], intervention studies are urgently needed to design and evaluate the efficacy of tailored resilience-building programs for this population. Importantly, beyond individual‑level interventions, future research should examine the impact of institutional policy changes, such as reforming evaluation systems, reducing publication pressure, or normalizing academic failure, on student mental health, using natural experimental or quasi‑experimental designs. Finally, qualitative research could provide deeper insights into the specific mechanisms through which resilience buffers against academic stress in the clinical training environment, and how trainees experience institutional support or adversity.

## Conclusions

This study shows that adverse academic events are positively associated with probable anxiety disorder among clinical medicine postgraduates. Crucially, resilience significantly moderates this relationship, corresponding to a substantially weaker association between adverse academic events and probable anxiety disorder. These findings underscore that fostering psychological resilience is not merely an adjunct to training but a critical component of developing sustainable and healthy medical professionals. Integrating systematic resilience assessment and resilience‑building programs into the fabric of postgraduate medical education should be a priority for institutions committed to trainee well-being.

## Data Availability

The datasets generated and analyzed during the current study are not publicly available due to restrictions specified in the ethical approval concerning participant confidentiality but are available from the corresponding author on reasonable request.

## Abbreviations

AAE: Adverse Academic Events
GAD-7: 7-item Generalized Anxiety Disorder Scale
CD-RISC-10: 10-item Connor-Davidson Resilience Scale
RERI: Relative Excess Risk due to Interaction
AP: Attributable Proportion
PR: Psychological Resilience
SI: Synergy Index
OR: Odds Ratio
CI: Confidence Interval

## References

[1] Forrester N. Mental health of graduate students sorely overlooked. Nature. 2021;595(7865):135–7. 34183825 10.1038/d41586-021-01751-z

[2] Woolston C. Stress and uncertainty drag down graduate students’ satisfaction. Nature. 2022;610(7933):805–8. 36280727 10.1038/d41586-022-03394-0

[3] Chi T, Cheng L, Zhang Z. Global prevalence and trend of anxiety among graduate students: A systematic review and meta-analysis. Brain Behav. 2023;13(4):e2909. 36852520 10.1002/brb3.2909 PMC10097092

[4] Ng IKS, Tham SZL, Chong KM, Goh WGW, Thong C, Teo KSH. Burnout among medical residents: key drivers and practical mitigating strategies. Postgrad Med J. 2025;101(1195):475–80. 39680959 10.1093/postmj/qgae179

[5] Zhang S, Li H, Zhou M, Chen J, Wang Q. Potential categories of academic stress and its relationship with positive academic emotions and social adaptation in medical postgraduates. Occupation Health. 2022;38(06):811–4.

[6] Balhatchet B, Schütze H, Williams N, Ashford B. Factors that impact burnout and psychological wellbeing in Australian postgraduate medical trainees: a systematic review protocol. Syst reviews. 2021;10(1):257. 10.1186/s13643-021-01809-z PMC8464131 34560887

[7] Jeyapalan T, Blair E. The Factors Causing Stress in Medical Students and their Impact on Academic Outcomes: A Narrative Qualitative Systematic Review. Int J Med Students. 2024;12(2):195–203.

[8] Obeng Nkrumah S, Adu MK, Agyapong B, da Luz Dias R, Agyapong VIO. Prevalence and correlates of depression, anxiety, and burnout among physicians and postgraduate medical trainees: a scoping review of recent literature. Front Public Health. 2025;13:1537108. 40697832 10.3389/fpubh.2025.1537108 PMC12279716

[9] Li Z, Wu M, Zhang X, Yan K, Wang X, Xu H, Li P, Liu Y, Deng Q, Li X, et al. Interrelationships of stress, burnout, anxiety, depression, quality of life and suicidality among Chinese residents under Standardized Residency Training: a network analysis. Ann Med. 2024;56(1):2433030. 39610267 10.1080/07853890.2024.2433030 PMC11610237

[10] Dyrbye LN, Harper W, Durning SJ, Moutier C, Thomas MR, Massie FS Jr., Eacker A, Power DV, Szydlo DW, Sloan JA, et al. Patterns of distress in US medical students. Med Teach. 2011;33(10):834–9. 21942482 10.3109/0142159X.2010.531158

[11] Karp JF, Levine AS. Mental Health Services for Medical Students - Time to Act. N Engl J Med. 2018;379(13):1196–8. 30257155 10.1056/NEJMp1803970

[12] Hazell CM, Chapman L, Valeix SF, Roberts P, Niven JE, Berry C. Understanding the mental health of doctoral researchers: a mixed methods systematic review with meta-analysis and meta-synthesis. Syst reviews. 2020;9(1):197. 10.1186/s13643-020-01443-1 PMC7450565 32847624

[13] Evans TM, Bira L, Gastelum JB, Weiss LT, Vanderford NL. Evidence for a mental health crisis in graduate education. Nat Biotechnol. 2018;36(3):282–4. 29509732 10.1038/nbt.4089

[14] Dyrbye LN, Thomas MR, Shanafelt TD. Systematic review of depression, anxiety, and other indicators of psychological distress among U.S. and Canadian medical students. Acad medicine: J Association Am Med Colleges. 2006;81(4):354–73. 10.1097/00001888-200604000-00009 16565188

[15] Huang PC, Lin CY, Huang RY, Chen JS, Potenza MN, Strong C, Wang HW, Griffiths MD, Chen CY, Ko NY, et al. Impact of COVID-19-Induced Academic Stress on Insomnia and Suicidal Ideation among Taiwanese Health Trainees and Junior Doctors. Med Sci monitor: Int Med J experimental Clin Res. 2024;30:e944932. 10.12659/MSM.944932 PMC11305106 38910318

[16] Yu M, Fu H, Xu H, Liu W, Wang C, Zhang P, Chi X. Current situation and causes of psychological stress in medical postgraduates. Med Educ Manage. 2025;11(03):360–5.

[17] WANG B, LAI W, LIU X, JIN M, LIU M, YAN Q, TAN Z, FU P. Mental health status and associated contributing factors among the medical students pursuing a professional master’s degree under thedual-track integrationtraining systems. West China Med J. 2024;39(07):1114–20.

[18] GAO Y, REN H, JI C. Determinants of mental health and construction of a risk prediction model a-mong clinical professional master’s students. J Harbin Med Univ. 2025;59(05):588–93.

[19] Sisto A, Vicinanza F, Campanozzi LL, Ricci G, Tartaglini D, Tambone V. Towards a transversal definition of psychological resilience: a literature review. Med (Kaunas Lithuania). 2019;55(11):745. 10.3390/medicina55110745 PMC6915594 31744109

[20] Masten AS. Ordinary magic. Resilience processes in development. Am Psychol. 2001;56(3):227–38. 11315249 10.1037//0003-066x.56.3.227

[21] Hobfoll SE. Conservation of resources. A new attempt at conceptualizing stress. Am Psychol. 1989;44(3):513–24. 2648906 10.1037//0003-066x.44.3.513

[22] Imran A, Tariq S, Kapczinski F, de Azevedo Cardoso T. Psychological resilience and mood disorders: a systematic review and meta-analysis. Trends psychiatry Psychother. 2024;46:e20220524. 36215270 10.47626/2237-6089-2022-0524 PMC11332678

[23] Southwick SM, Bonanno GA, Masten AS, Panter-Brick C, Yehuda R. Resilience definitions, theory, and challenges: interdisciplinary perspectives. Eur J Psychotraumatol. 2014;5:1–14. 10.3402/ejpt.v5.25338 PMC4185134 25317257

[24] Zhou Y, Gao W, Li H, Yao X, Wang J, Zhao X. Network analysis of resilience, anxiety and depression in clinical nurses. BMC Psychiatry. 2024;24(1):719. 39438840 10.1186/s12888-024-06138-8 PMC11520162

[25] Liu S, Lu J, He Y, Zheng G, Liu S, Xiang Y, Wang X, Liu X, Xiao Y. The sequential association between depression, anxiety, and functional constipation among Chinese children and adolescents: A mediation by resilience. J Affect Disord. 2025;390:119845. 40639541 10.1016/j.jad.2025.119845

[26] Tüscher O. Neurobiological mechanisms of psychological resilience - a conceptual framework: Oliver Tüscher. Eur J Pub Health. 2017;27(suppl_3):185.

[27] Xu S, Zhang Q. Coping styles, resilience, and mental health symptoms in first-year medical students: a structural equation modeling approach in a cross-sectional study. BMC Psychol. 2025;13(1):1229. 41194301 10.1186/s40359-025-03561-8 PMC12590866

[28] Abend R. Understanding anxiety symptoms as aberrant defensive responding along the threat imminence continuum. Neurosci Biobehav Rev. 2023;152:105305. 37414377 10.1016/j.neubiorev.2023.105305 PMC10528507

[29] Fredrickson BL. The role of positive emotions in positive psychology. The broaden-and-build theory of positive emotions. Am Psychol. 2001;56(3):218–26. 11315248 10.1037//0003-066x.56.3.218 PMC3122271

[30] Breakwell GM. Identity resilience: its origins in identity processes and its role in coping with threat. Contemp Social Sci. 2021;16(5):573–88.

[31] Paersch C, Schulz A, Wilhelm FH, Brown AD, Kleim B. Recalling autobiographical self-efficacy episodes boosts reappraisal-effects on negative emotional memories. Emot (Washington DC). 2022;22(6):1148–58. 10.1037/emo0000949 33630625

[32] Spitzer RL, Kroenke K, Williams JB, Löwe B. A brief measure for assessing generalized anxiety disorder: the GAD-7. Arch Intern Med. 2006;166(10):1092–7. 16717171 10.1001/archinte.166.10.1092

[33] Tong X, An D, McGonigal A, Park SP, Zhou D. Validation of the Generalized Anxiety Disorder-7 (GAD-7) among Chinese people with epilepsy. Epilepsy Res. 2016;120:31–6. 26709880 10.1016/j.eplepsyres.2015.11.019

[34] Connor KM, Davidson JR. Development of a new resilience scale: the Connor-Davidson Resilience Scale (CD-RISC). Depress Anxiety. 2003;18(2):76–82. 12964174 10.1002/da.10113

[35] Yu XN, Lau JT, Mak WW, Zhang J, Lui WW, Zhang J. Factor structure and psychometric properties of the Connor-Davidson Resilience Scale among Chinese adolescents. Compr Psychiatry. 2011;52(2):218–24. 21295229 10.1016/j.comppsych.2010.05.010

[36] Knol MJ, VanderWeele TJ. Recommendations for presenting analyses of effect modification and interaction. Int J Epidemiol. 2012;41(2):514–20. 22253321 10.1093/ije/dyr218 PMC3324457

[37] Botto LD, Khoury MJ. Commentary: facing the challenge of gene-environment interaction: the two-by-four table and beyond. Am J Epidemiol. 2001;153(10):1016–20. 11384958 10.1093/aje/153.10.1016

[38] Andersson T, Alfredsson L, Källberg H, Zdravkovic S, Ahlbom A. Calculating measures of biological interaction. Eur J Epidemiol. 2005;20(7):575–9. 16119429 10.1007/s10654-005-7835-x

[39] Menacho-Rivera J, Castro-Ramirez L, Yarasca-Berrocal E, Huamani-Echaccaya J, Hernández-Vergara C, Ladera-Castañeda M, Cayo-Rojas C. Academic burnout syndrome associated with anxiety, stress, depression, and quality of life in Peruvian dentistry students: an analysis using a multivariable regression model. BMC Med Educ. 2025;25(1):998. 40611047 10.1186/s12909-025-07604-x PMC12225186

[40] Lugassy D, Ben-Izhack G, Zissu S, Shitrit Lahav R, Rosner O, Elzami R, Shely A, Naishlos S. Anxiety, stress, and depression levels among dental students: gender, age, and stage of dental education related. Psychol health Med. 2025;30(7):1394–408. 40105006 10.1080/13548506.2025.2476085

[41] Gao X, Zeng S, Huang Y, Hao C, Xiang X, Meng S, Wang L, Yang X, Janak LP, Liu Y, et al. Emotion regulation as a mediator between stress and burnout in dental postgraduate students: a South China cross-sectional study. BMC Med Educ. 2025;25(1):1663. 41316217 10.1186/s12909-025-08262-9 PMC12664252

[42] Otukoya EZ, Amiri A, Alimohammadi E. Surgeon well-being: a systematic review of stressors, mental health, and resilience. BMC Surg. 2025;25(1):430. 41044709 10.1186/s12893-025-03180-5 PMC12495620

[43] Jeamjitvibool T, Duangchan C, Mousa A, Mahikul W. The association between resilience and psychological distress during the COVID-19 pandemic: a systematic review and meta-analysis. Int J Environ Res Public Health. 2022;19(22):1–17. 10.3390/ijerph192214854 PMC9690093 36429573

[44] Hobfoll SE, Halbesleben J, Neveu J-P, Westman M. Conservation of Resources in the Organizational Context: The Reality of Resources and Their Consequences. 5 2018. 2018;5:103–28.

[45] Wadi M, Shorbagi A, Shorbagi S, Taha MH, Bahri Yusoff MS. The impact of the Systematic Assessment for Resilience (SAR) framework on students’ resilience, anxiety, depression, burnout, and academic-related stress: a quasi-experimental study. BMC Med Educ. 2024;24(1):506. 38715022 10.1186/s12909-024-05444-9 PMC11077819

[46] AM K, I H, A JK. Psychological interventions to foster resilience in healthcare students. Cochrane Database Syst Reviews. 2020;7:CD013684. 10.1002/14651858.CD013684 PMC7388680 32691879

